# Supplementary material for: Biomechanical regulation of breast cancer metastasis and progression
Source: Sci Rep. 2021 May 10;11:9838. doi: 10.1038/s41598-021-89288-z (PMC8110548; doi:10.1038/s41598-021-89288-z)
Supplement: Supplementary file 1 — Supplementary information. [file 41598_2021_89288_MOESM1_ESM.pdf]

Supplemental Materials for

Biomechanical Regulation of Breast Cancer Metastasis and Progression

Adrianne Spencer[1†], Andrew D. Sligar[1†], Daniel Chavarria[1], Jason Lee[1], Darshil Choksi[1], Nikita P. Patil[1], HooWon Lee[1], Austin P. Veith[1], William J. Riley[1], Shubh Desai[1], Ali Abbaspour[1], Rohan Singeetham[1], and Aaron B. Baker[1,2,3,4*]

[1]Department of Biomedical Engineering, University of Texas, Austin, TX

[2]Institute for Cellular and Molecular Biology, University of Texas at Austin, Austin, TX

[3]Institute for Biomaterials, Drug Delivery and Regenerative Medicine, University of Texas at Austin, Austin, TX

[4]Institute for Computational Engineering and Sciences, University of Texas at Austin, Austin, TX

[*]Correspondence to:

Aaron B. Baker

University of Texas at Austin

Department of Biomedical Engineering

1 University Station

BME 5.202D, C0800

Austin, TX 78712

Phone: 512-232-7114

E-mail: abbaker1@gmail.com

Supplemental Figure Legends

Supplemental Figure 1. Mechanical strain regulates gene expression in functional gene groups. MDA-MB-231 breast cancer cells were mechanically strained at 0, 7.5 or 15% strain at a frequency of 1 Hz for 24 hours (n = 4). (A) Volcano plots for comparisons between the groups. (B-E) RNA sequencing was performed and gene groups were examined including genes associated with drug metabolism, proliferation, Yap/Zeb transcriptional control, and cell adhesion. *$p < 0.05$ versus static group. †$p < 0.05$ versus 7.5% strain group. R software was used to create parts of this figure [23].

Supplemental Figure 2. Mechanical strain reduces metabolic activity in cancer cells. MDA-MB-231 cells were treated with 7.5% mechanical strain for 24 hours and metabolic activity was measured using an MTT assay.

Supplemental Figure 3. Mechanical strain reduces growth and enhances chemoresistance of MCF-7 cells. MCF-7 cells were cells were treated with 7.5% mechanical strain (1 Hz) for 24 hours with treatment with doxorubicin at the indicated amount (n = 13-16). *$p < 0.05$ versus the static group under treatment with the same concentration of doxorubicin. †$p < 0.05$ versus the static group with 0 μg/ml doxorubicin.

Supplemental Figure 4. Mechanical strain does not induce changes in multidrug resistance protein activity in MDA-MB-231 breast cancer cells. MDA-MB-231 breast cancer cells were mechanically strained for 24 hours at 0 or 7.5% strain. The cells were then tested for activity of multidrug resistance proteins MDR1, MRP1, and BCRP. Flow cytometry histogram of cell counts for gold dye intensity in (A) statically cultured cells and (B) cells conditioned with 7.5% cyclic mechanical strain. Increasing presence of multidrug resistance efflux pumps would decrease concentration of gold dye inside the cell. (C) Multidrug resistance activity factor was calculated to determine the influence of three multidrug resistance efflux pumps, multi drug resistance protein 1 (MDR1), MDR-associated protein (MRP1), and breast cancer resistance protein (BCRP).

Supplemental Figure 5. Immunoblotting for signaling pathway activation by mechanical load. MDA-MB-231 were treated with 7.5% cyclic strain for the time indicated, lysed and immunoblotted for the signaling pathway activation.

Supplemental Figure 6. Full western blot gels utilized in Supplemental Figure 5. MDA-MB-231 were treated with 7.5% cyclic strain for the time indicated, lysed and immunoblotted for the signaling pathway activation.

Supplemental Figure 7. Full western blot gels utilized in Figure 2E. Experimental groups are, starting from left to right, static cells treated with DMSO, asciminib, radotinib, AZD1480, ruxolitinib and PI3K inhibitor followed by the cells treated with 7.5% strain DMSO, asciminib, radotinib, AZD1480, ruxolitinib, and PI3K inhibitor.

Supplemental Figure 8. Full western blot gels utilized in Figure 2F. Experimental groups are, starting from left to right, static cells treated with DMSO, asciminib, radotinib, AZD1480, ruxolitinib and PI3K inhibitor followed by the cells treated with 7.5% strain DMSO, asciminib, radotinib, AZD1480, ruxolitinib, and PI3K inhibitor.

Supplemental Figure 9. Summary diagram of mechanisms inferred by studies on mechanically induced enhancement in cell survival and chemoresistance.

Supplemental Figure 10. Mechanical strain increases adhesion of MCF-7 cells to endothelial cells and specific ECM. MCF-7 cells were mechanically loaded for 24 hours at 7.5% strain at 1 Hz. Initial adhesion of strained cells under 0.5 dynes/cm^2 shear stress to a endothelial monolayer with or without pre-treatment with TNF-α or to ECM coated plates. *$p < 0.05$ compared to the static group (n = 7-16).

Supplemental Figure 11. Mechanical strain increases actin stress fiber formation. MDA-MB-231 cells were mechanically loaded for 24 hours at 7.5% strain at 1 Hz and then stained for F-actin. *$p < 0.05$ compared to the static group (n = 5-6). Scale bar = 20 μm.

Supplemental Figure 12. Vasculogenic activity is increased by mechanical loading in breast cancer cells. MBA-MB-231 cells were mechanically loaded for 24 hours at 7.5% strain at 1 Hz. (A) Endothelial cells were treated with conditioned media from mechanically loaded cancer cells in tube formation assay on matrigel. $^{*}p < 0.05$ versus static group. (B) MDA-MB-231 cells were treated with mechanical load and then used directly in a tube formation assay on matrigel. $^{*}p < 0.05$ versus static group.

Supplemental Figure 13. Full western blot gels utilized in Figure 5E. Experimental groups are, starting from left to right, static cells treated with just cell media, DMSO, Lck inhibitor, PI3K inhibitor, Syk inhibitor, and Yap inhibitor (Vert; verteporfin), followed by the cells treated with 7.5% strain cell media, DMSO, Lck inhibitor, PI3K inhibitor, Syk inhibitor, and Yap inhibitor (Vert).

Supplemental Figure 14. Full western blot gels utilized in Figure 5F. Experimental groups are, starting from left to right, static cells treated with just cell media, DMSO, Lck inhibitor, PI3K inhibitor, Syk inhibitor, and Yap inhibitor (Vert; verteporfin), followed by the cells treated with 7.5% strain cell media, DMSO, Lck inhibitor, PI3K inhibitor, Syk inhibitor, and Yap inhibitor (Vert).

Supplemental Figure 15. Full western blot gels utilized in Figure 5G. Experimental groups are, starting from left to right, static cells treated with just cell media, DMSO, Lck inhibitor, PI3K inhibitor, Syk inhibitor, and Yap inhibitor (Vert; verteporfin), followed by the cells treated with 7.5% strain cell media, DMSO, Lck inhibitor, PI3K inhibitor, Syk inhibitor, and Yap inhibitor (Vert).

Supplemental Figure 16. Full western blot gels utilized in Figure 5H. Experimental groups are, starting from left to right, static cells treated with just cell media, DMSO, Lck inhibitor, PI3K inhibitor, Syk inhibitor, and Yap inhibitor (Vert; verteporfin), followed by the cells treated with 7.5% strain cell media, DMSO, Lck inhibitor, PI3K inhibitor, Syk inhibitor, and Yap inhibitor (Vert).

Supplemental Figure 17. Full western blot gels utilized in Figure 5I. Experimental groups are, starting from left to right, static cells treated with just cell media, DMSO, Lck inhibitor, PI3K inhibitor, Syk inhibitor, and Yap inhibitor (Vert; verteporfin), followed by the cells treated with 7.5% strain cell media, DMSO, Lck inhibitor, PI3K inhibitor, Syk inhibitor, and Yap inhibitor (Vert).

Supplemental Figure 18. Full western blot gels utilized in Figure 5J. Experimental groups are, starting from left to right, static cells treated with just cell media, DMSO, Lck inhibitor, PI3K inhibitor, Syk inhibitor, and Yap inhibitor (Vert; verteporfin), followed by the cells treated with 7.5% strain cell media, DMSO, Lck inhibitor, PI3K inhibitor, Syk inhibitor, and Yap inhibitor (Vert).

Supplemental Figure 19. Tumor volumes for orthotopic implantation study. (A) Tumor volumes for tumors at three days. (B) Full time course of tumor volumes for the study.

Supplemental Figure 20. Tumor weights for excised tumors from the orthotopic implantation study.

Supplemental Figure 21. Laser speckle imaging for late time point in orthotopic tumor model. MDA-MB-231 cells were treated with mechanical load for 24 hours and then injected into the mammary fat pad of nu/nu mice. (A) Laser speckle images of the mice following treatment. The dashed circles illustrate the location of the mammary fat pad. The right mammary fat pad received the injection and the left served as a contralateral control. (B) Quantification of the relative perfusion of the injected fat pad to the non-injected fat pad.

Supplemental Figure 22. Micro-CT images of the skulls from mice injected with MDA-MB-231 cells grown under static conditions or 7.5% strain for 24 hours.

Supplemental Figure 1

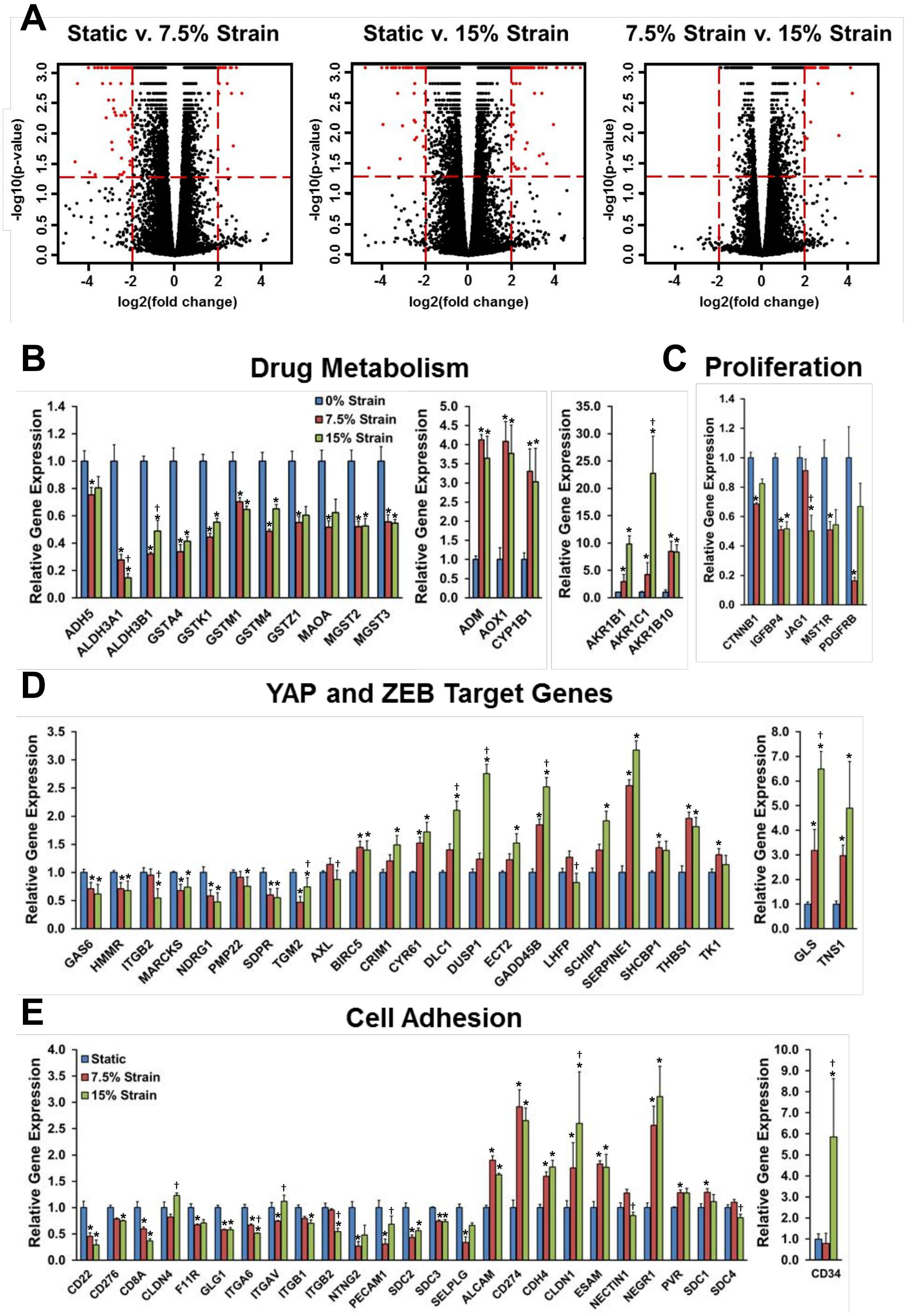

Supplemental Figure 2

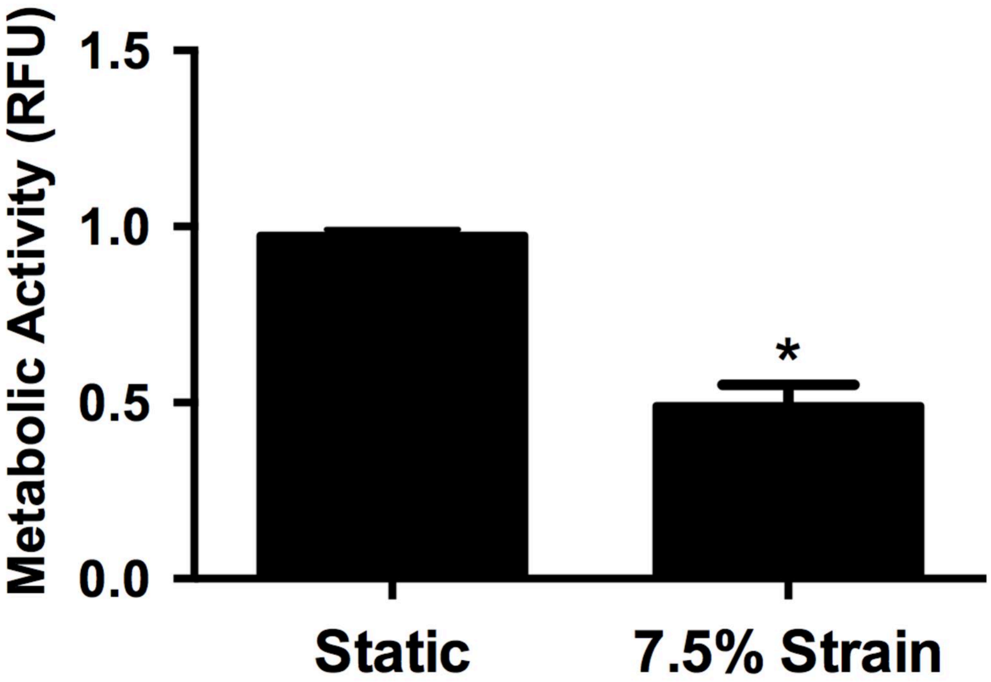

Supplemental Figure 3

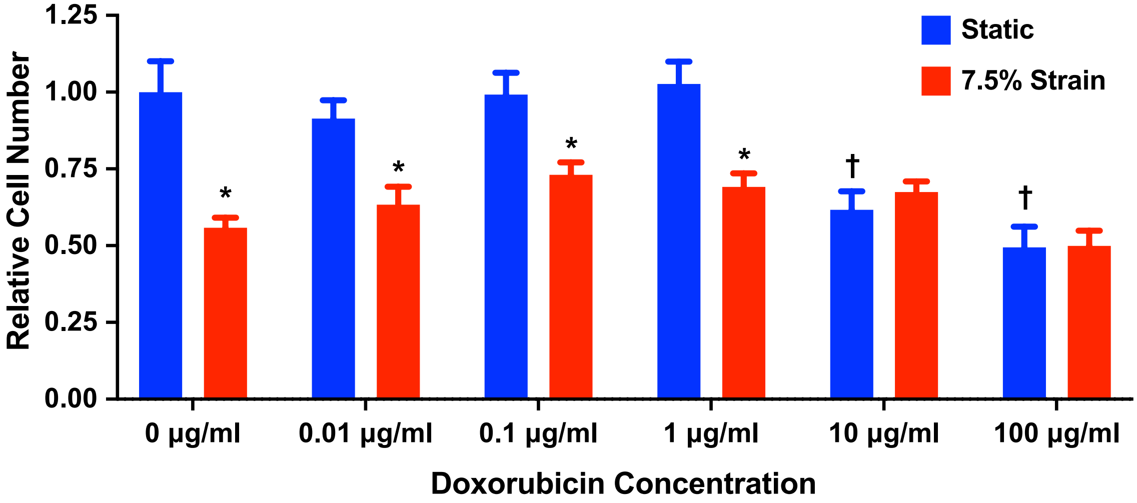

Supplemental Figure 4

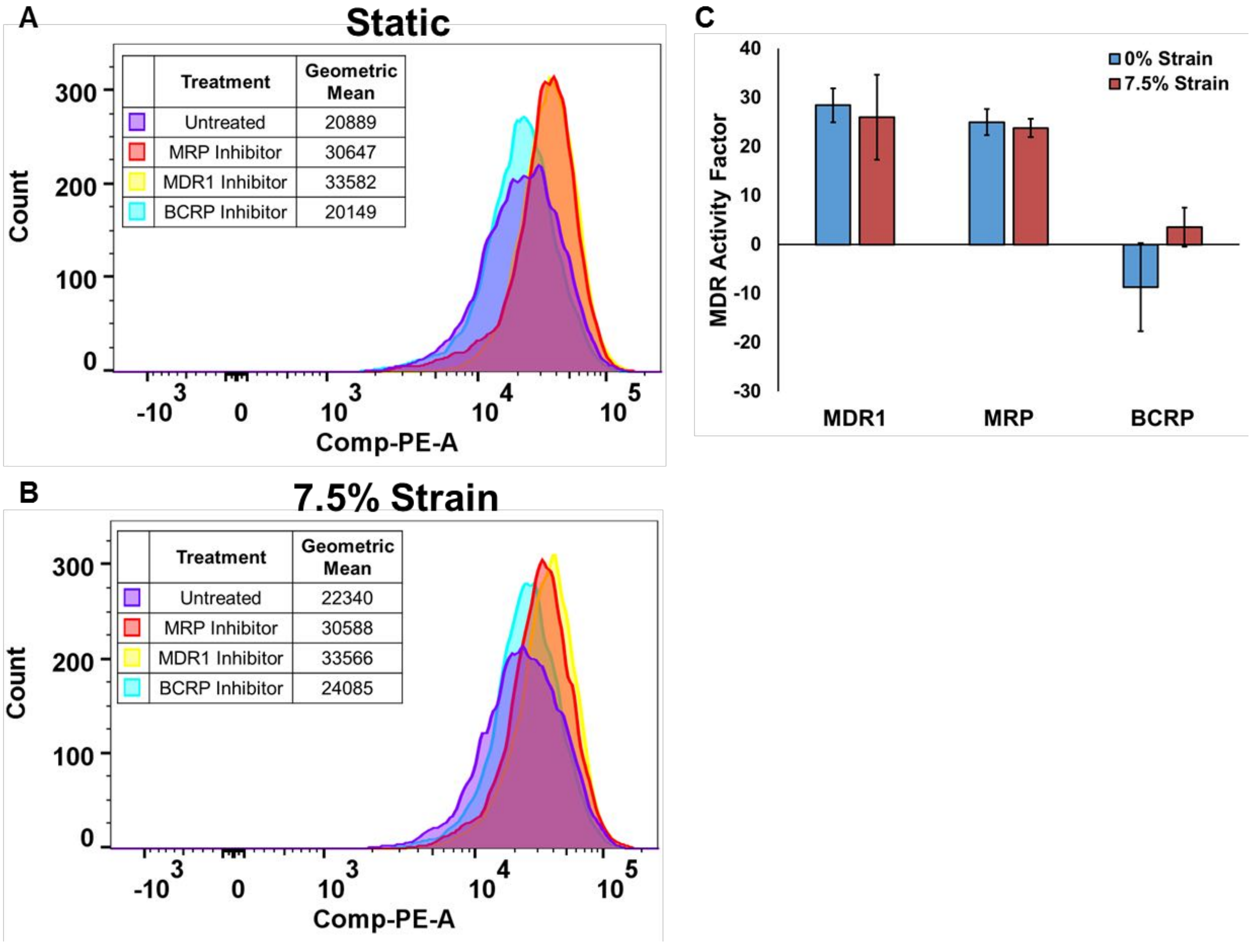

Supplemental Figure 5

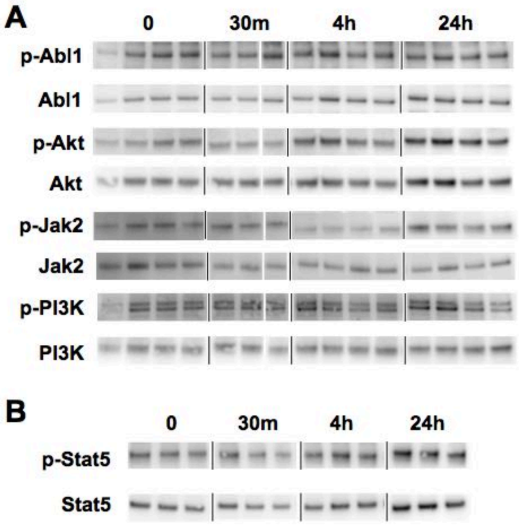

Supplemental Figure 6

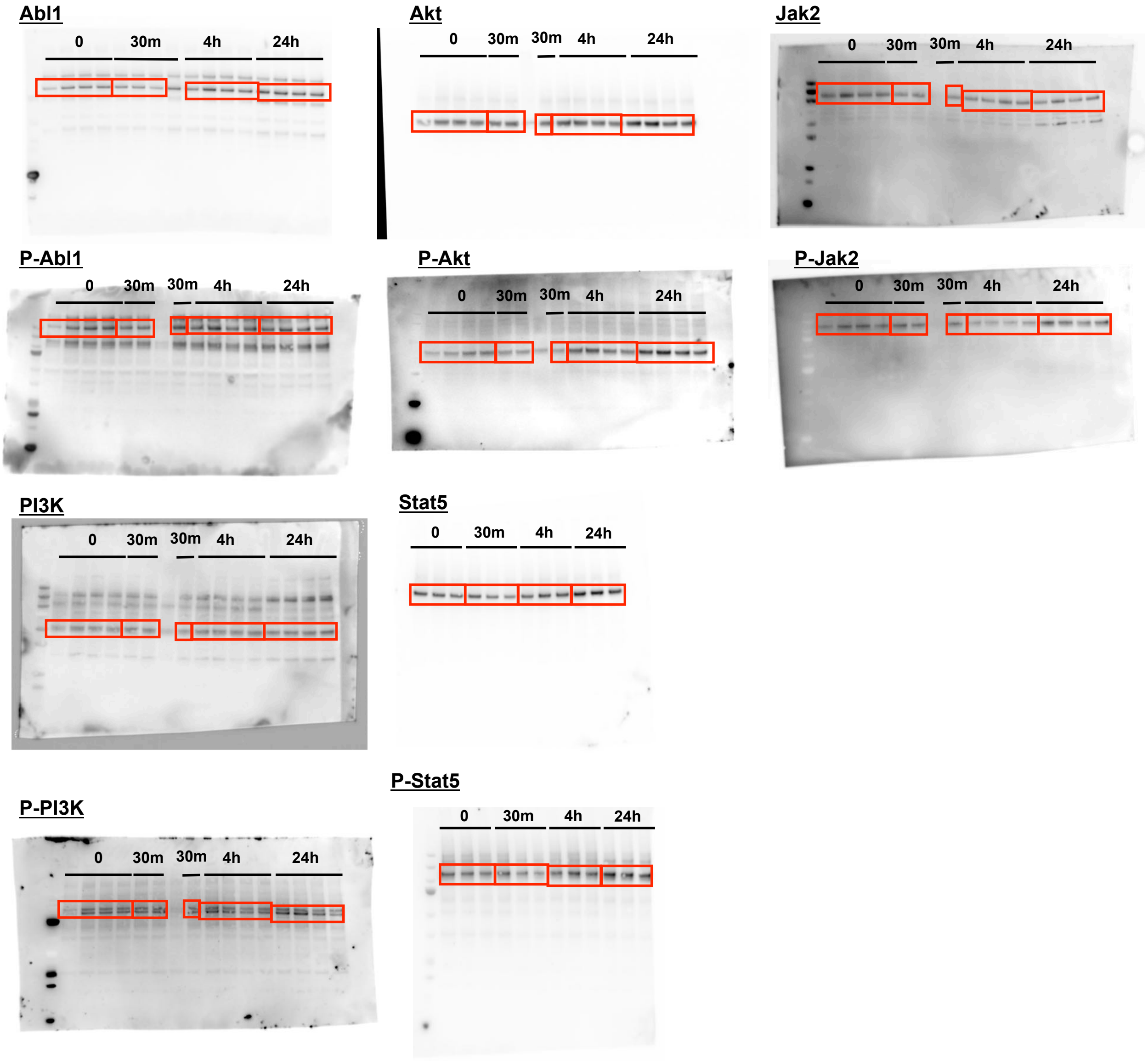

Supplemental Figure 7

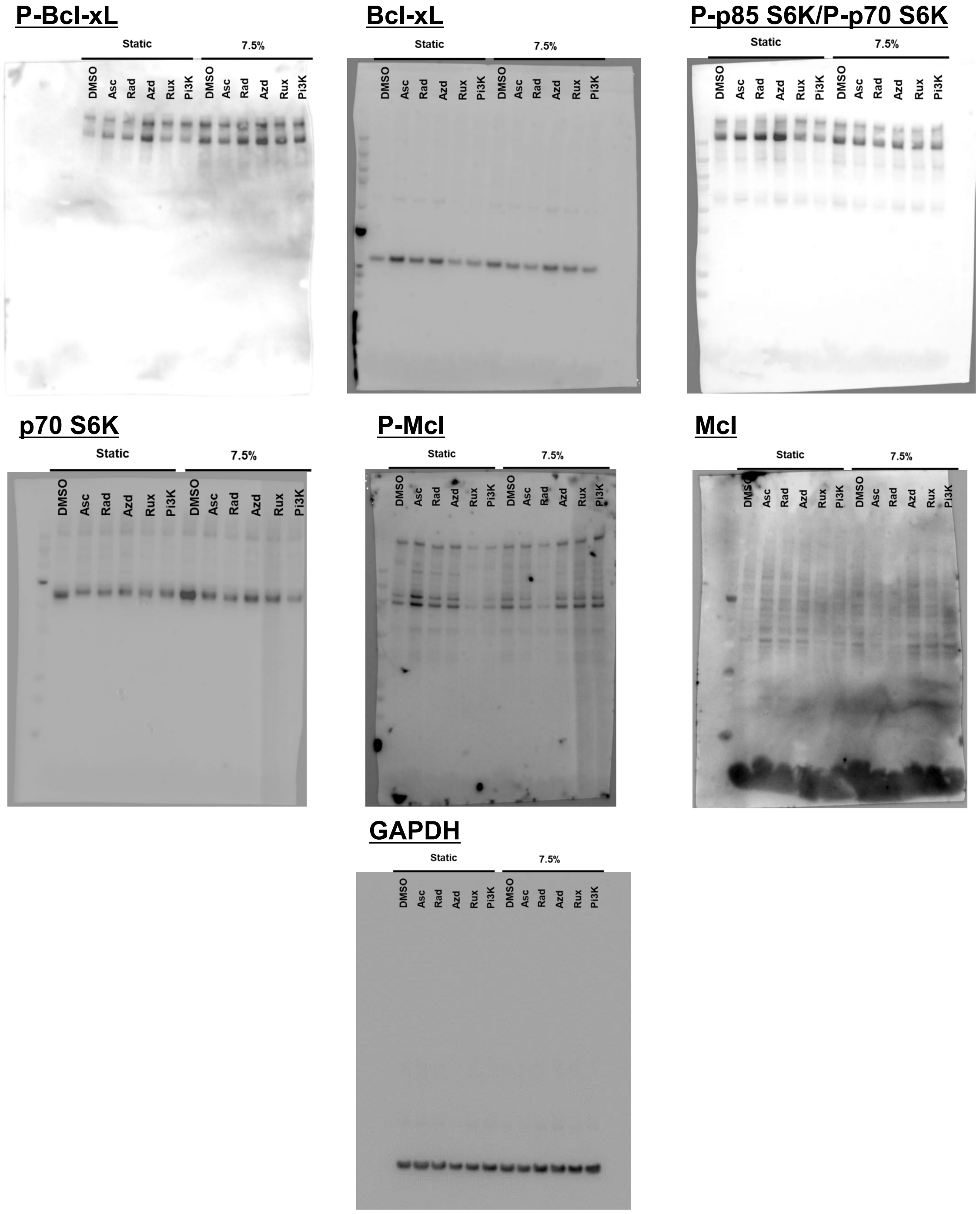

Supplemental Figure 8

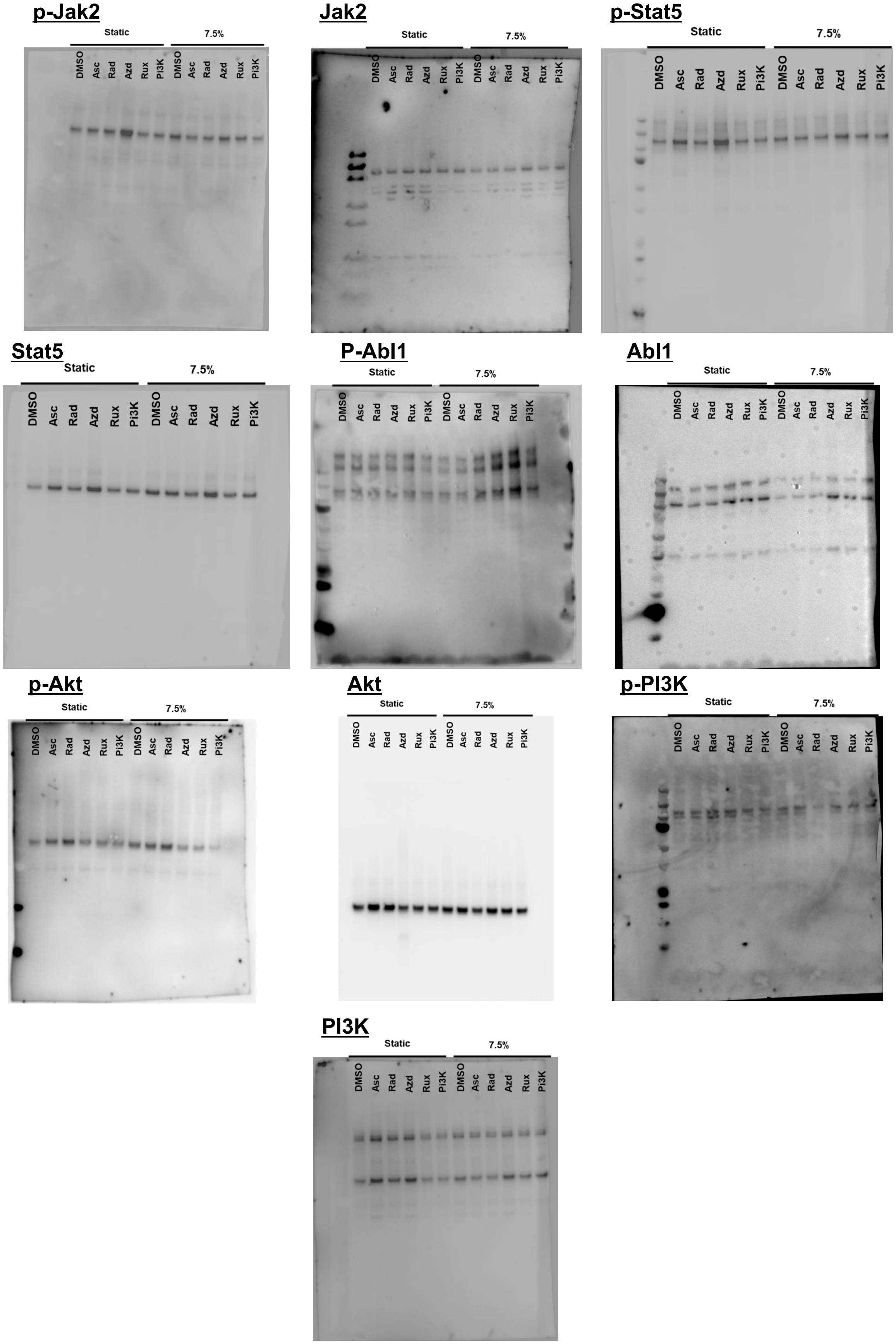

Supplemental Figure 9

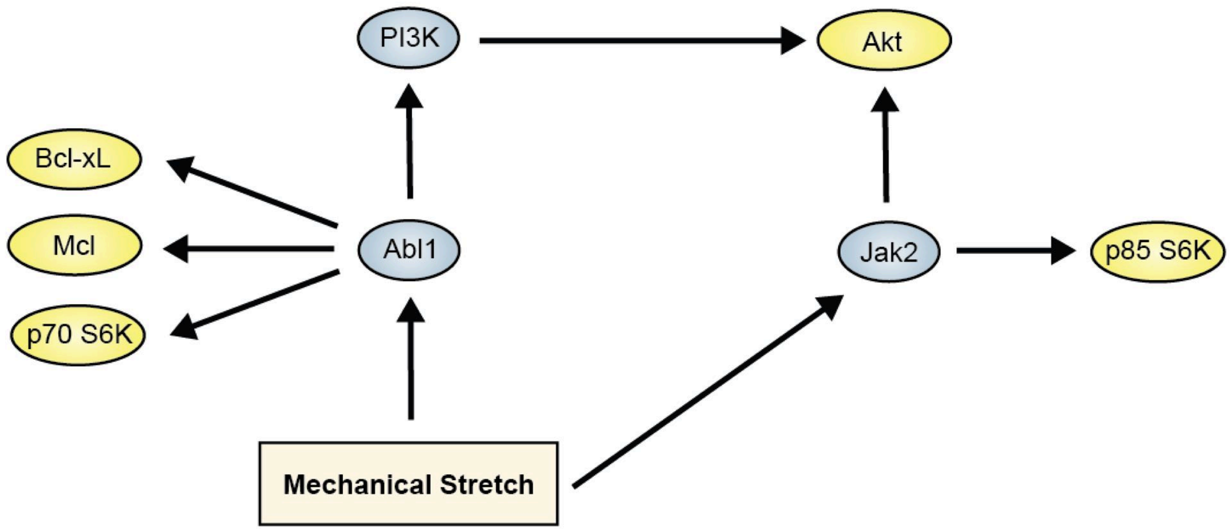

Supplemental Figure 10

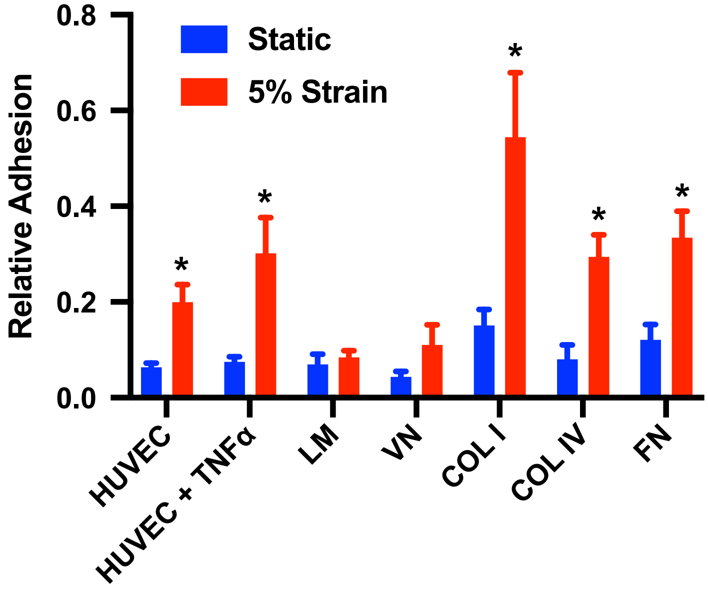

Supplemental Figure 11

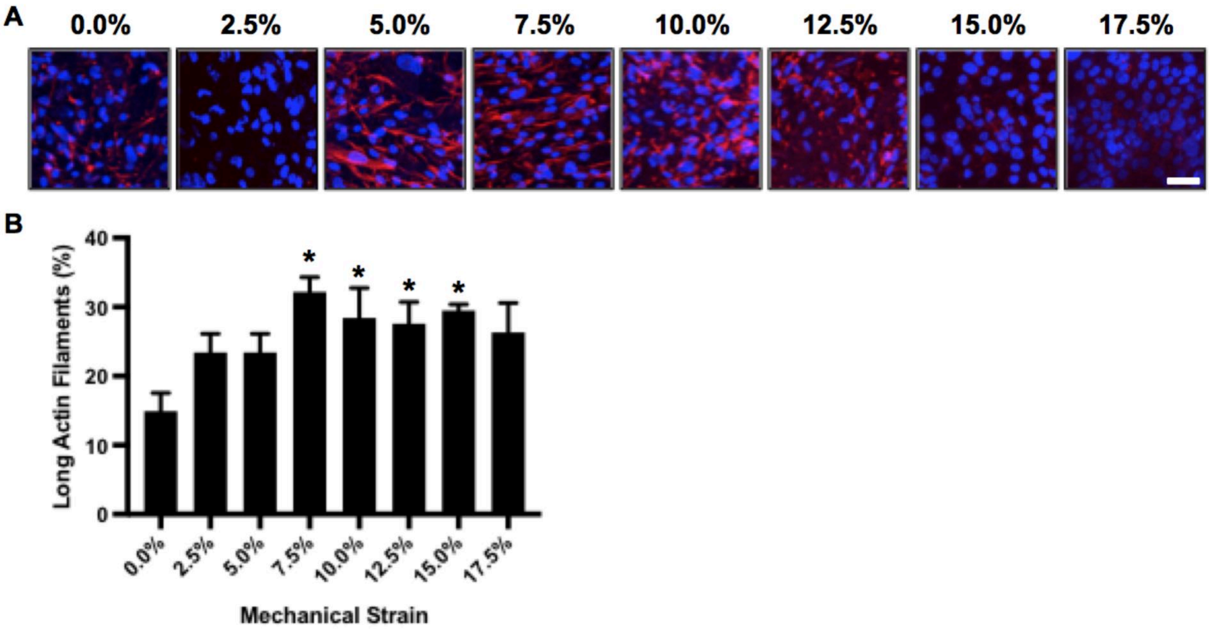

Supplemental Figure 12

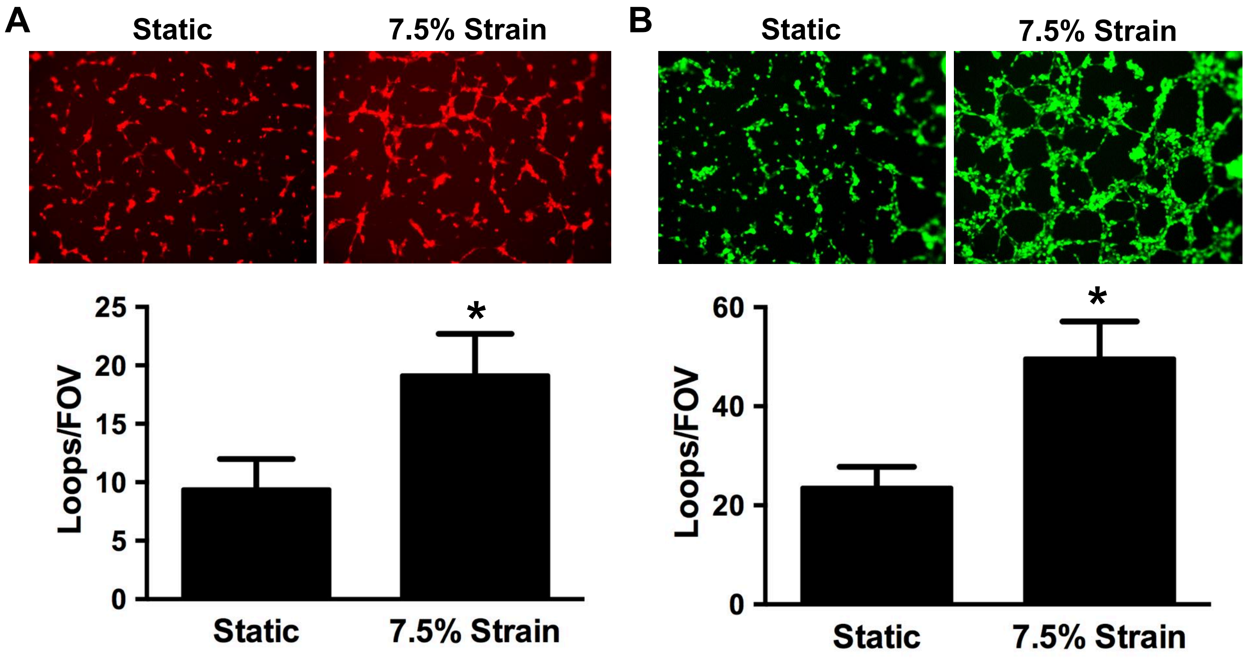

Supplemental Figure 13

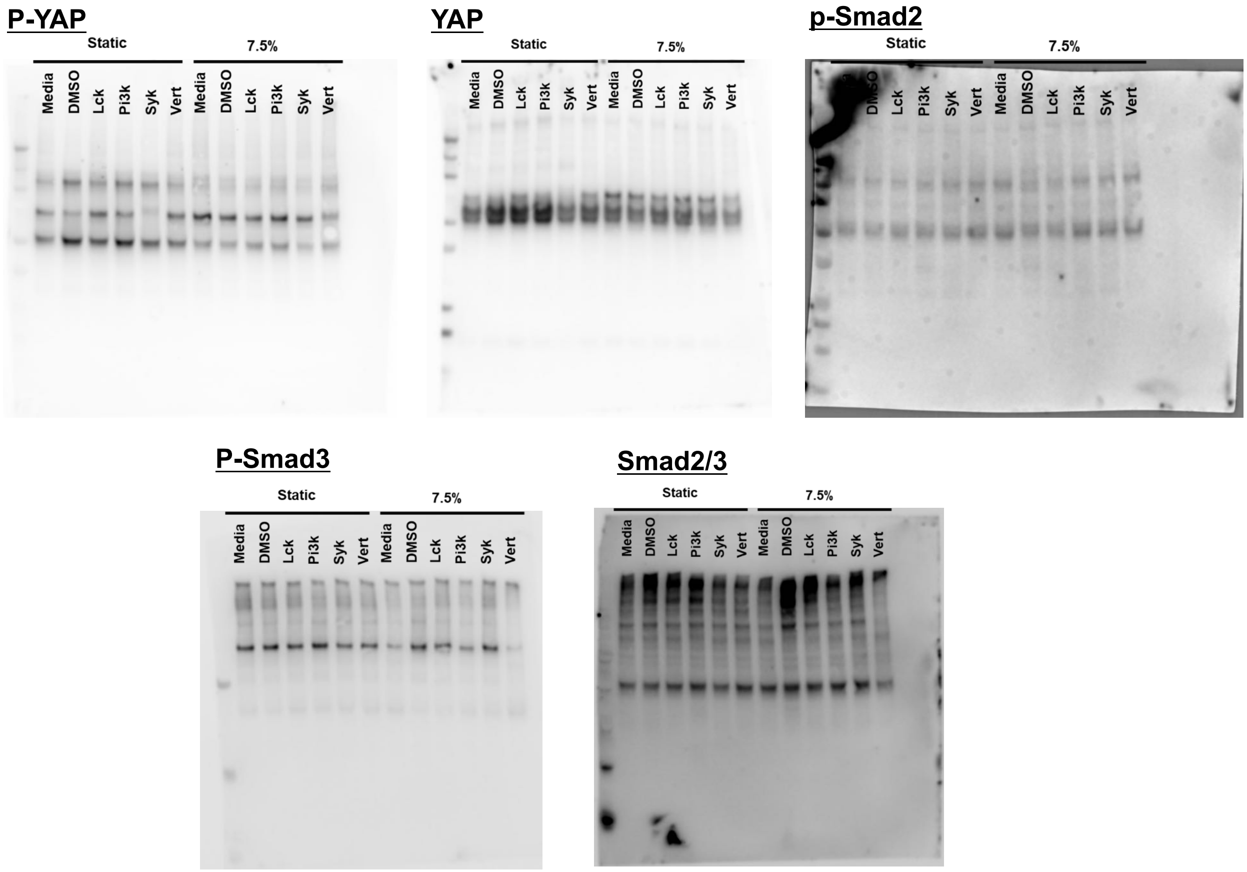

Supplemental Figure 14

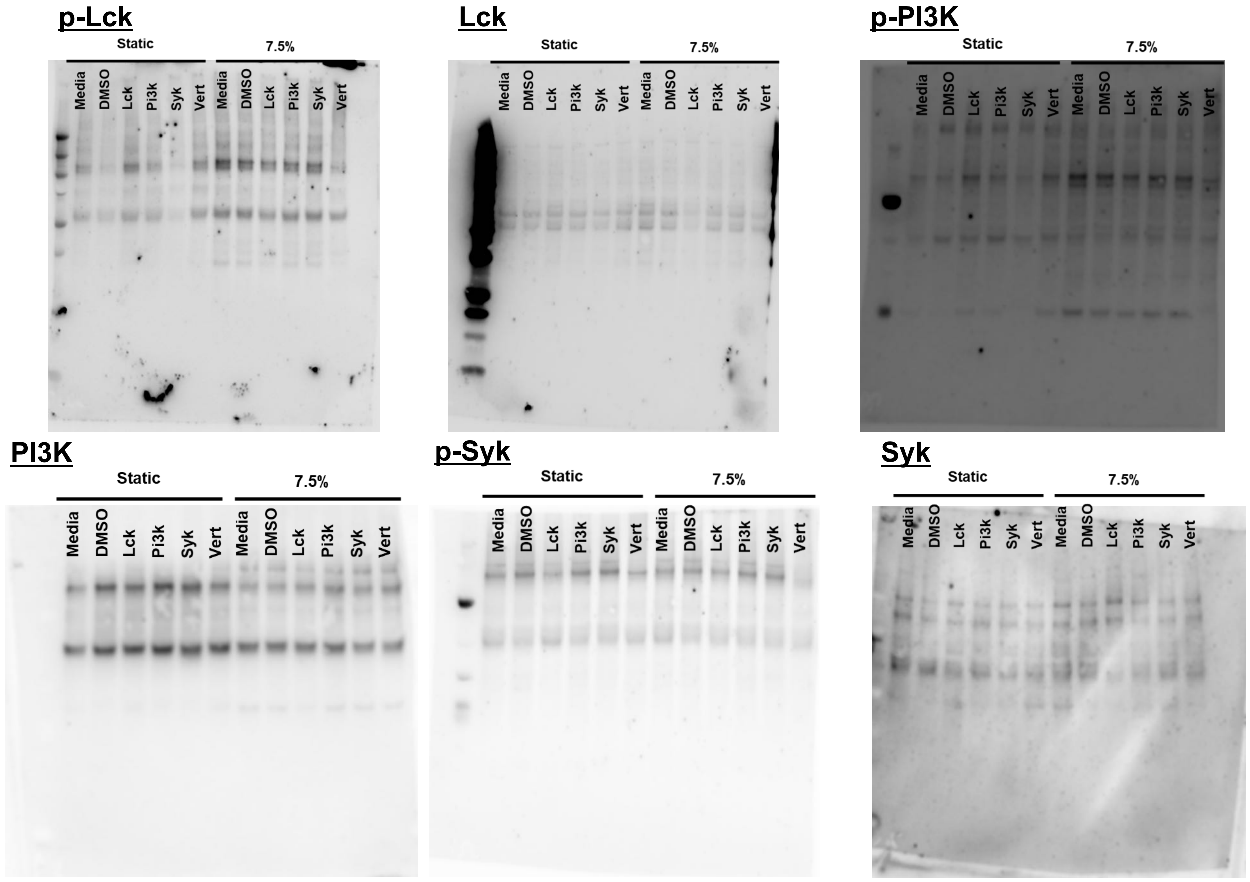

Supplemental Figure 15

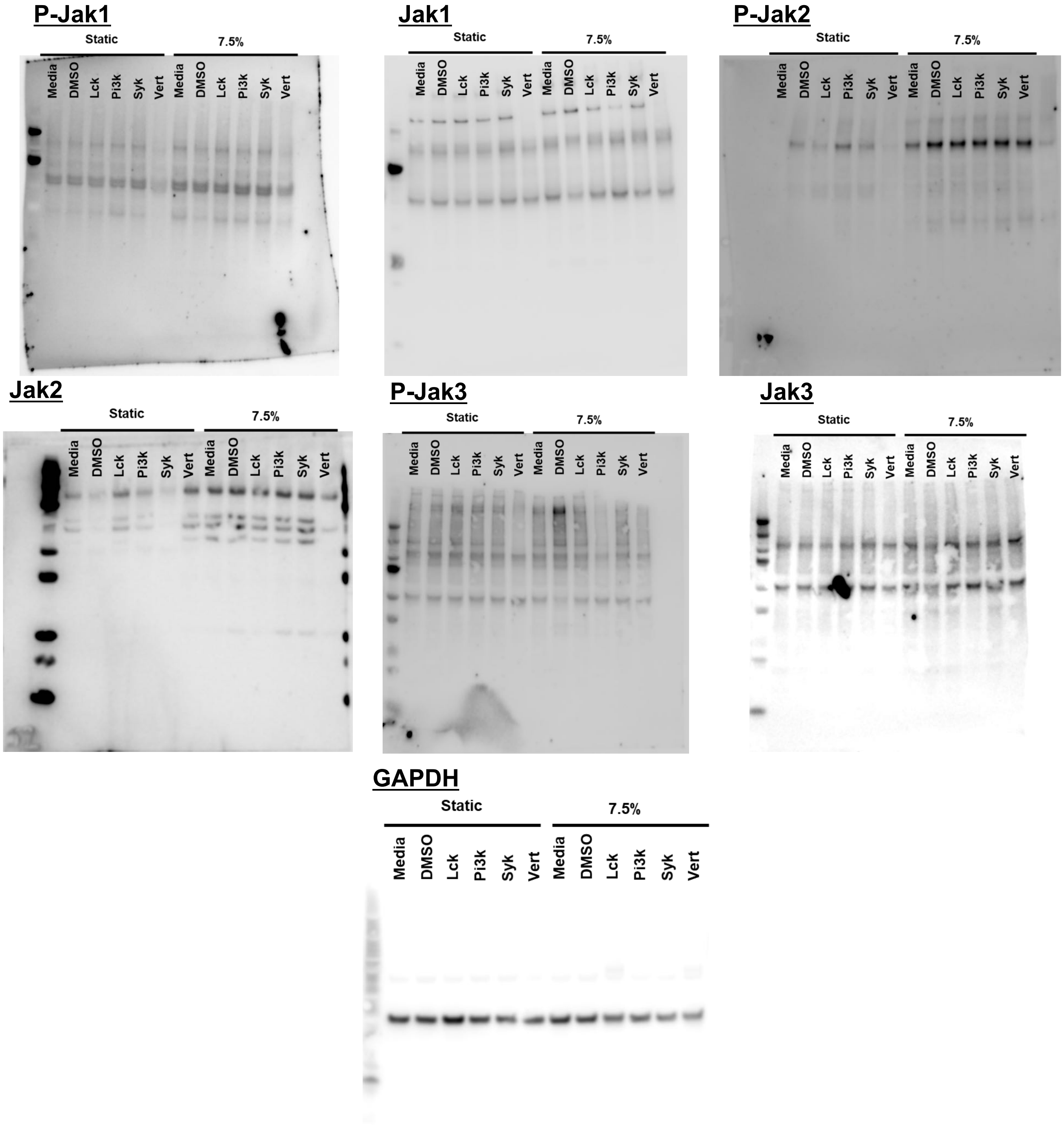

Supplemental Figure 16

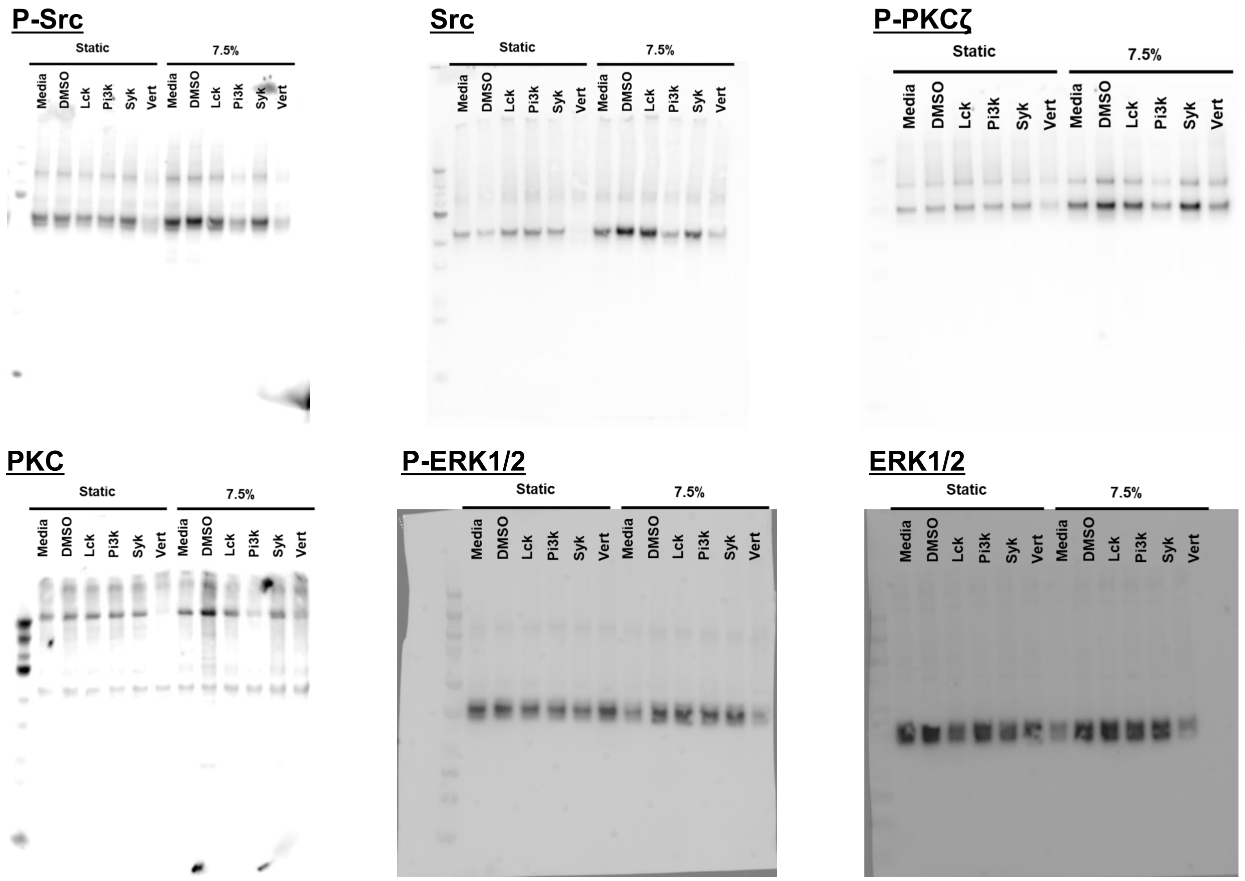

Supplemental Figure 17

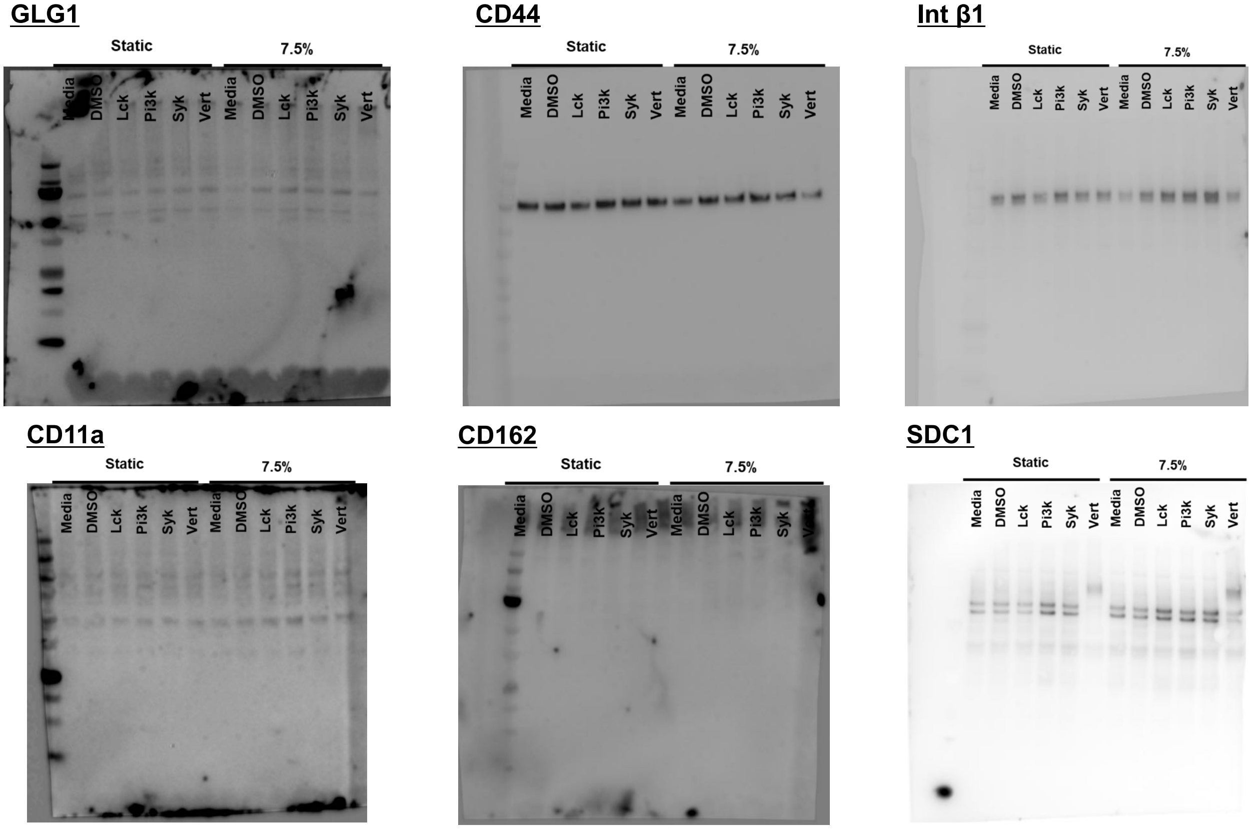

Supplemental Figure 18

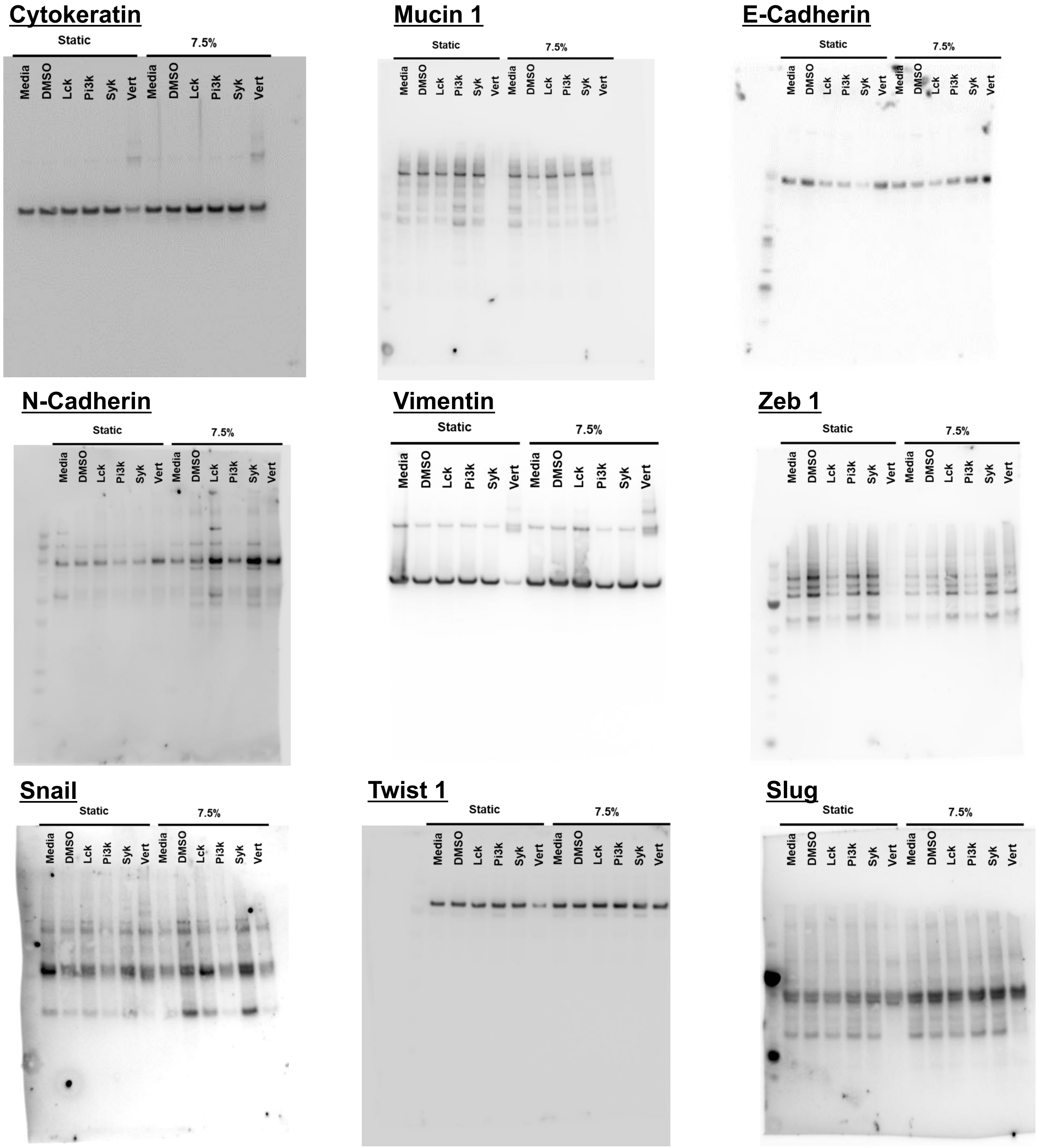

Supplemental Figure 19

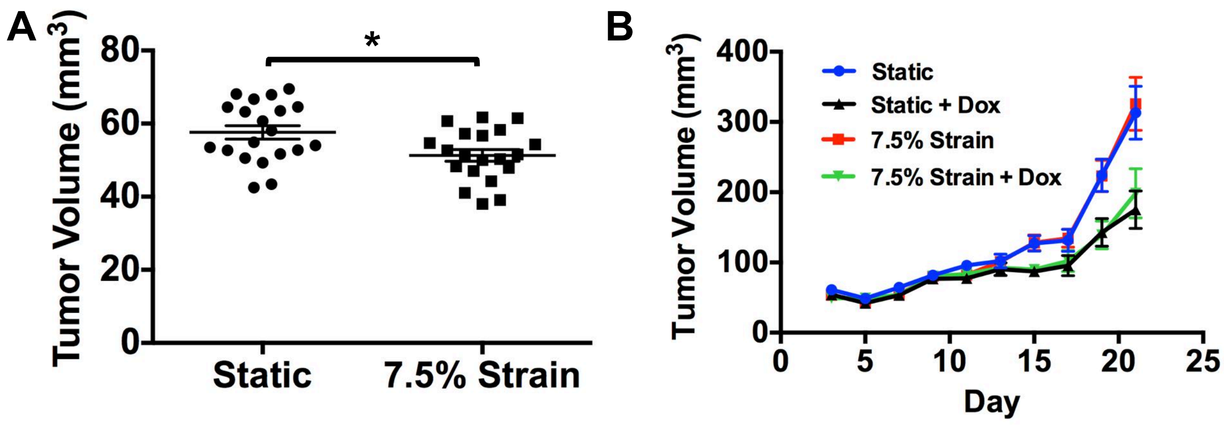

Supplemental Figure 20

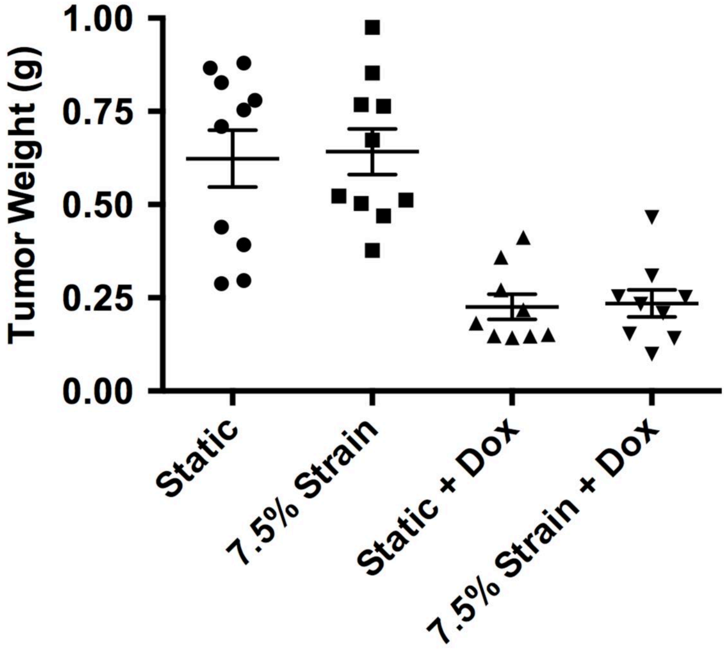

Supplemental Figure 21

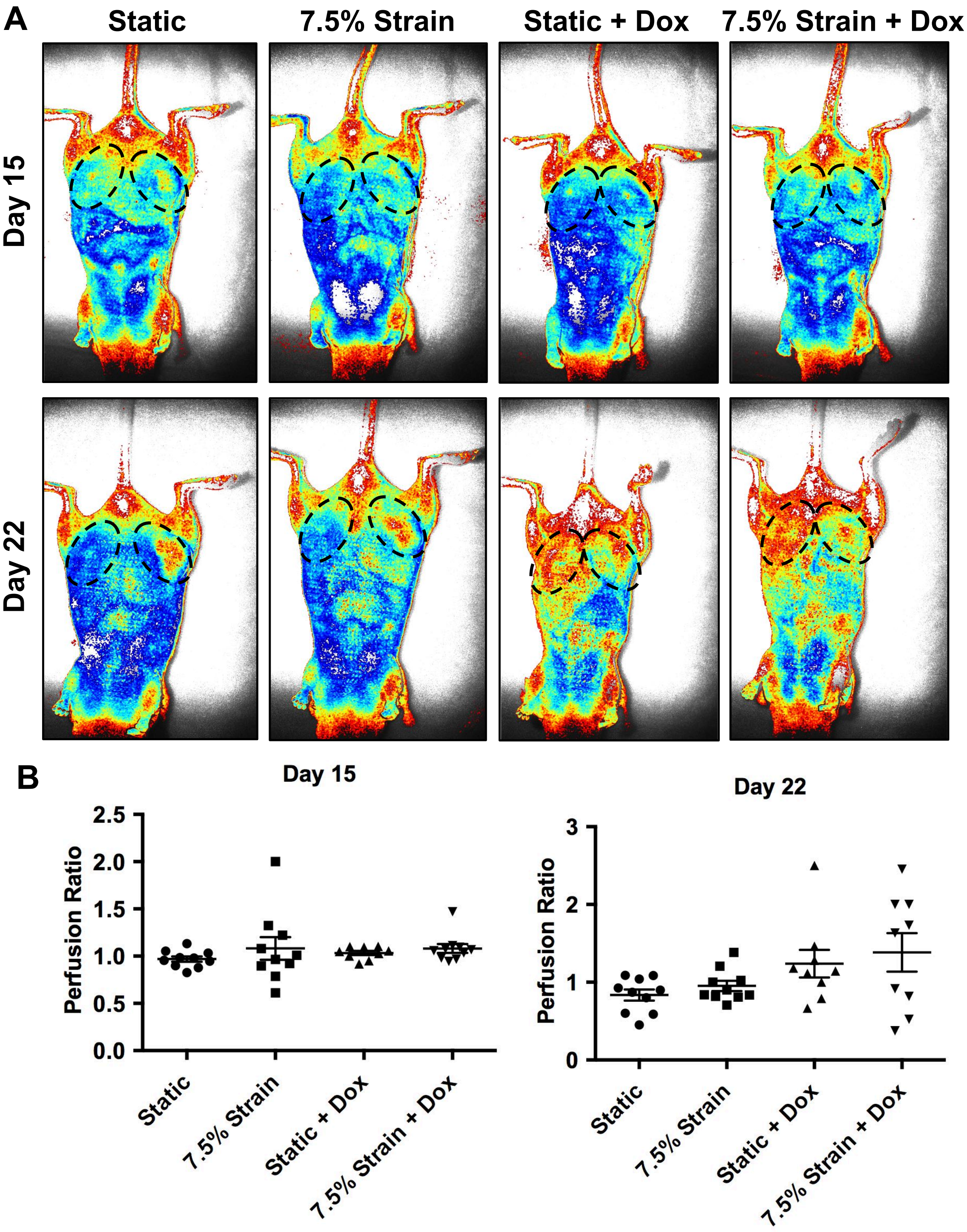

Supplemental Figure 22

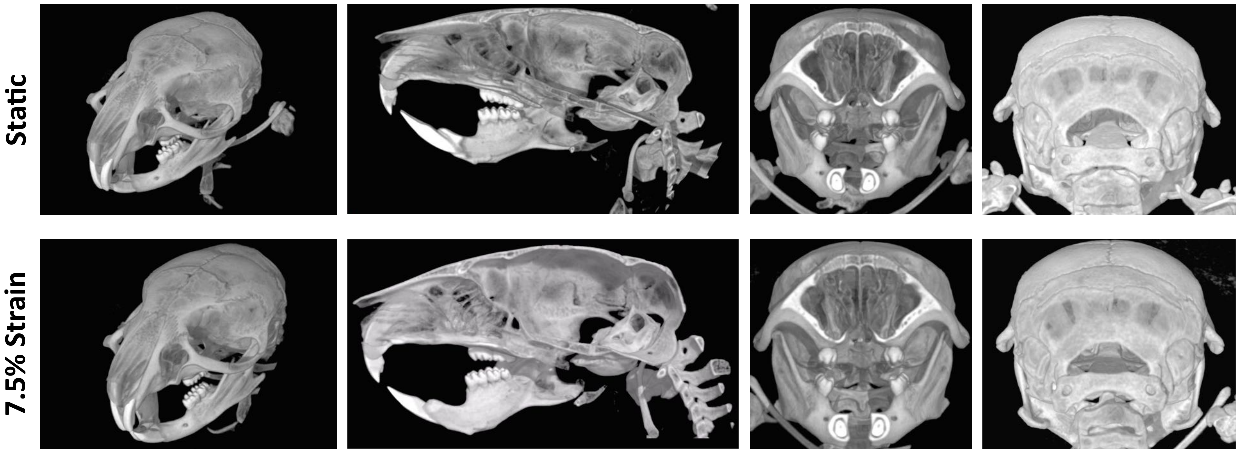

Supplemental Tables

Supplemental Table 1. ***Integrin inhibitors Used in the Study.***

Drug	Target
ATN-161	$\alpha5\beta1$
BIO-1211	$\alpha4\beta1$
Cilengitide	$\alpha v\beta3$ and $\alpha v\beta5$
Obtustatin	$\alpha1\beta1$
P11	$\alpha5\beta1$/vitronectin
PF-562271	FAK
RGDS	Pan-integrin
TC-I 15	$\alpha5\beta1$

Supplemental Table 2. *Primary Antibodies/Reagents Used for Immunostaining*

Target Protein	Company	Catalog #	Species/Isotype	Dilution Ratio
AF 594 Phalloidin	Invitrogen	A12381	N/A	1:250
Paxillin	Santa Cruz Biotechnology	sc-5574	anti-rabbit	1:50
PECAM-1	Cell Signaling	3528S	anti-mouse	1:100
α-SMA	Abcam	ab21027	anti-rabbit	1:100
ILK	BD Biosciences	611803	anti-mouse	1:100
Yap/Taz	Cell Signaling	8418S	anti-rabbit	1:100
14-3-3ε	Santa Cruz Biotechnology	sc393177	anti-mouse	1:50
Smad 2/3	BD Biosciences	610842	anti-mouse	1:100
Phospho-Smad2/3	Cell Signaling	8828S	anti-rabbit	1:100
vWF	Santa Cruz Biotechnology	365712	anti-mouse	1:50
Myosin IIb	Cell Signaling	3404	anti-rabbit	1:100
Sca-1	Santa Cruz Biotechnology	sc-365343	anti-mouse	1:50
Oct-4	Cell Signaling	2750	anti-rabbit	1:100

Supplemental Table 3. ***Antibodies Used in the Studies.***

Target	Dilution	Product #	Supplier	Method
**14-3-3ε**	1:50	sc-393177	Santa Cruz Biotechnology	ICC
**Abl**	1:250	ab15130	Abcam	WB
**Akt**	1:250	4685	Cell Signaling Technology	WB
**Bax**	1:100	ab53154	Abcam	ICC
**Bcl-2**	1:50	sc-7382	Santa Cruz Biotechnology	ICC
**Bcl-xl**	1:250	2762	Cell Signaling Technology	WB
**CD11a**	1:250	ab52895	Abcam	WB
**CD162**	1:250	MAB996	R&D Systems	WB
**CD166**	1:20	562936	BD Biosciences	Flow Cytometry
**CD274**	1:20	563742	BD Biosciences	Flow Cytometry
**CD29**	1:20	743786	BD Biosciences	Flow Cytometry
**CD325**	1:20	563434	BD Biosciences	Flow Cytometry
**CD44**	1:20	744271	BD Biosciences	Flow Cytometry
**CD44**	1:250	37259	Cell Signaling Technology	WB
**CD51/CD61**	1:20	744088	BD Biosciences	Flow Cytometry
**Claudin-1**	1:20	FAB4618R	R&D Systems	Flow Cytometry
**Cyclin D1**	1:250	2978	Cell Signaling Technology	WB
**E-Cadherin**	1:50	sc-7870	Santa Cruz Biotechnology	ICC. WB
**E-Cadherin**	1:20	FAB18381G	R&D Systems	Flow Cytometry
**Erk**	1:250	9102	Cell Signaling Technology	WB
**ESAM**	1:20	FAB4204G	R&D Systems	Flow Cytometry
**GAPDH**	1:500	2118	Cell Signaling Technology	WB
**GLG1**	1:250	PA528987	Thermo Fisher Scientific	WB
**Hamster IgG HRP**	1:3500	PA132045	Thermo Fisher Scientific	WB
**Int β1**	1:250	4706	Cell Signaling Technology	WB
**Jak1**	1:250	3332	Cell Signaling Technology	WB
**Jak2**	1:250	3230	Cell Signaling Technology	WB
**Jak3**	1:250	3775	Cell Signaling Technology	WB
**Ki-67**	1:100	9449S	Cell Signaling Technology	ICC
**Lck**	1:250	2752	Cell Signaling Technology	WB
**Mcl**	1:250	4572	Cell Signaling Technology	WB
**Mouse IgG Alexa 594**	1:500	ab150108	Abcam	ICC
**Mouse IgG HRP**	1:3500	sc-2318	Santa Cruz Biotechnology	WB
**Mouse IgG HRP**	1:3500	AP192P	EMD Millipore	WB
**N-Cadherin**	1:100	sc-7939	Santa Cruz Biotechnology	ICC. WB
**p70 S6k**	1:250	2708	Cell Signaling Technology	WB
**pAbl1**	1:250	ab4717	Abcam	WB
**pAkt**	1:250	9271	Cell Signaling Technology	WB
**pBcl-xl**	1:250	44428G	Thermo Fisher Scientific	WB
**pCyclin D1**	1:250	3300	Cell Signaling Technology	WB
**pErk**	1:250	9101	Cell Signaling Technology	WB
**Pi3k**	1:250	11889	Cell Signaling Technology	WB
**pJak1**	1:250	3331	Cell Signaling Technology	WB
**pJak2**	1:250	3771	Cell Signaling Technology	WB

**pJak3**	1:250	5031	Cell Signaling Technology	WB
**PKC**	1:250	PA536757	Thermo Fisher Scientific	WB
**pLck**	1:250	2751	Cell Signaling Technology	WB
**pMcl**	1:250	4579	Cell Signaling Technology	WB
**pp70/p85 S6k**	1:250	9204	Cell Signaling Technology	WB
**pPi3k**	1:250	4228	Cell Signaling Technology	WB
**pPKCζ**	1:250	2060	Cell Signaling Technology	WB
**pSmad2**	1:250	3108	Cell Signaling Technology	WB
**pSmad2/3**	1:100	8828	Cell Signaling Technology	ICC. WB
**pSmad3**	1:250	9520	Cell Signaling Technology	WB
**pSrc**	1:250	2101	Cell Signaling Technology	WB
**pSyk**	1:250	2711	Cell Signaling Technology	WB
**pYap**	1:250	13008	Cell Signaling Technology	WB
**Rabbit IgG 488**	1:500	ab150073	Abcam	ICC
**Rabbit IgG HRP**	1:3500	A16104	Thermo Fisher Scientific	WB
**Rabbit IgG HRP**	1:3500	sc-2077	Santa Cruz Biotechnology	WB
**Slug**	1:100	9585S	Cell Signaling Technology	ICC. WB
**Smad2/3**	1:50	sc-133098	Santa Cruz Biotechnology	ICC. WB
**Smad2/3**	1:250	3102	Cell Signaling Technology	WB
**Snail-1**	1:100	sc-271977	Santa Cruz Biotechnology	ICC. WB
**Src**	1:250	2108	Cell Signaling Technology	WB
**Syk**	1:250	2712	Cell Signaling Technology	WB
**Syndecan-1**	1:250	12922	Cell Signaling Technology	WB
**VE-Cadherin**	1:100	AF1002	R&D Systems	ICC. WB
**Vimentin**	1:50	sc-5565	Santa Cruz Biotechnology	ICC. WB
**vWF**	1:100	sc-365712	Santa Cruz Biotechnology	ICC. WB
**Yap/Taz**	1:100	8418S	Cell Signaling Technology	ICC
**Yap/Taz**	1:250	4912	Cell Signaling Technology	WB
**α−SMA**	1:100	ab5694	Abcam	ICC. WB
**β-Catenin**	1:50	sc-7199	Santa Cruz Biotechnology	ICC. WB
